# [Corrigendum] Effects of matrine on the proliferation and apoptosis of vincristine-resistant retinoblastoma cells

**DOI:** 10.3892/etm.2025.13040

**Published:** 2025-12-04

**Authors:** Bowen Zhao, Bin Li, Qian Liu, Fei Gao, Zhibao Zhang, Haixia Bai, Yichen Wang

Exp Ther Med 20:2838–2844, 2020; DOI: 10.3892/etm.2020.8992

Following the publication of the above paper, it was drawn to the Editor's attention by an interested reader that, regarding Fig. 4 on p. 2841, four of the flow cytometric plots portrayed in this figure that were supposedly describing cell cycle arrest at G_0_/G_1_ phase following treatment with matrine for 0, 12, 24 and 48 h appeared to be identical with data shown in Fig. 3 in an article featuring some of the same authors that had appeared several years previously in the journal *Investigative Ophthalmology & Visual Science*, although the experiments were reported to be different.

Upon contacting the authors, they realized that the incorrect set of data had been included in the above paper (in spite of the time that had elapsed between the publication of this paper and their former study, the experiments for both articles were performed at around the same time). A revised version of Fig. 4, featuring the data from one of the repeated sets of experiments, is shown below. All the authors agree to the publication of this Corrigendum, and are grateful to the Editor of *Experimental and Therapeutic Medicine* for granting them the opportunity to publish this; moreover, they apologize to the readership for any inconvenience caused.

## Figures and Tables

**Figure 4:**
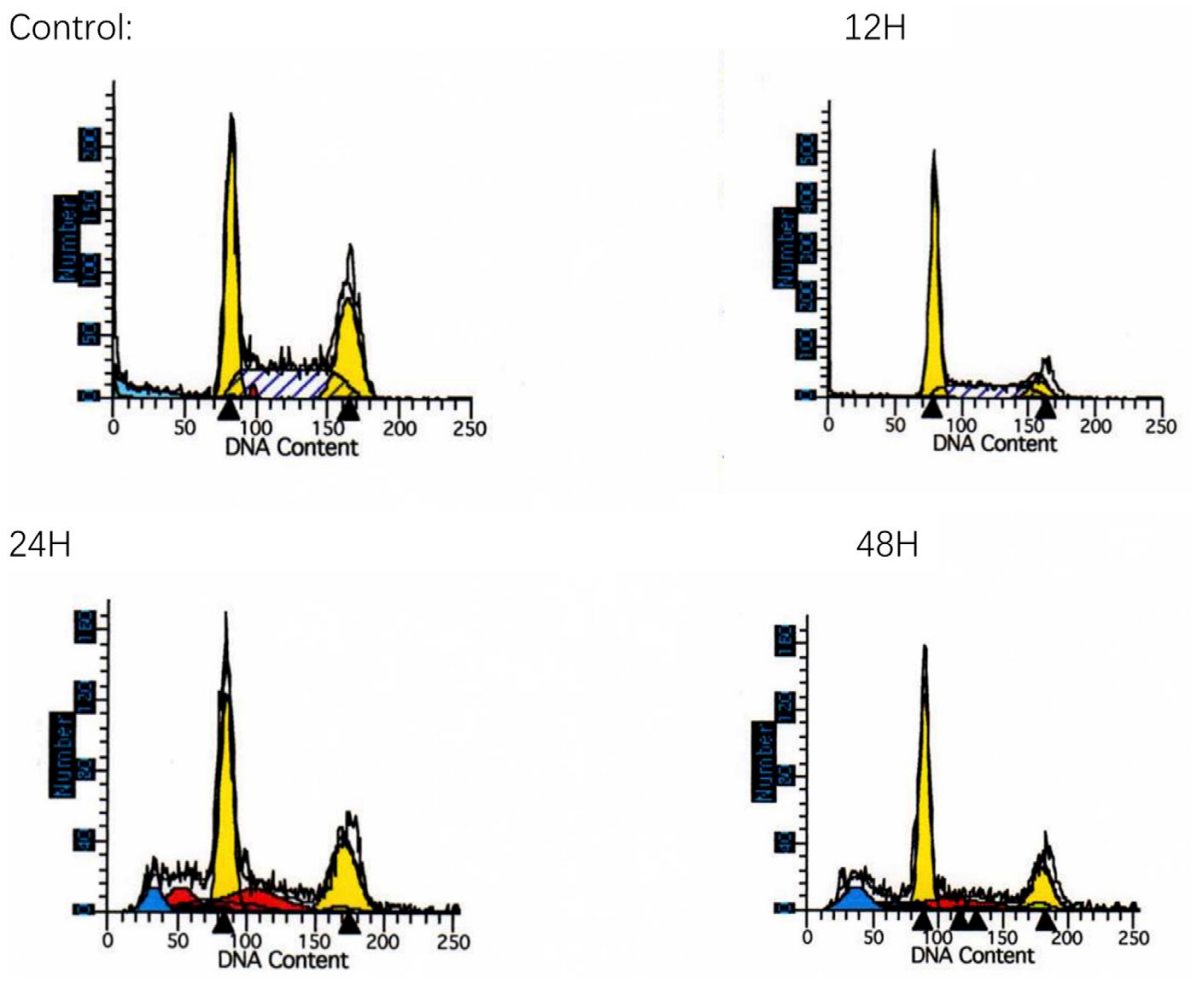
Matrine arrests the cell cycle at G0/G1 phase. After treatment with matrine for 0, 12, 24 and 48 h, cell cycle was arrested at G0/G1 phase. Arrows indicate cells at the G1 and G2 phase, respectively.

